# State-of-the-art monitoring in treatment of dengue shock syndrome: a case series

**DOI:** 10.1186/s13256-016-1019-z

**Published:** 2016-08-24

**Authors:** Steven L. Moulton, Jane Mulligan, Anon Srikiatkhachorn, Siripen Kalayanarooj, Greg Z. Grudic, Sharone Green, Robert V. Gibbons, Gary W. Muniz, Carmen Hinojosa-Laborde, Alan L. Rothman, Stephen J. Thomas, Victor A. Convertino

**Affiliations:** 1 Department of Surgery, University of Colorado, School of Medicine, 12631 E. 17th Avenue, C-305, Aurora, CO 80045 USA; 2 Flashback Technologies, Inc., 7490 Clubhouse Rd, Boulder, CO 80301 USA; 3 Department of Medicine, University of Massachusetts Medical School, 55 N Lake Ave, Worcester, MA 01655 USA; 4 Queen Sirikit National Institute for Child Health Hospital, 420/8 Ratchawithi Road, Thung Phaya Thai, Khet Ratchathewi, Bangkok, 10400 Thailand; 5 Armed Forces Research Institute of Medical Sciences, 315/6 Rajvithi Road, Bangkok, 10400 Thailand; 6 US Army Institute of Surgical Research, 3698 Chambers Pass, JBSA Fort Sam Houston, TX 78234-7549 USA; 7 Department of Cell and Molecular Biology, University of Rhode Island, 393 CBLS, 120 Flagg Road, Kingston, Rhode Island 02881 USA; 8 Viral Diseases, Walter Reed Army Institute of Research, 503 Robert Grant Ave, Silver Spring, MD 20910 USA

**Keywords:** Machine learning, Decision support, Pulse oximetry, Dengue shock syndrome

## Abstract

**Background:**

Early recognition and treatment of circulatory volume loss is essential in the clinical management of dengue viral infection. We hypothesized that a novel computational algorithm, originally developed for noninvasive monitoring of blood loss in combat casualties, could: (1) indicate the central volume status of children with dengue during the early stages of “shock”; and (2) track fluid resuscitation status.

**Methods:**

Continuous noninvasive photoplethysmographic waveforms were collected over a 5-month period from three children of Thai ethnicity with clinical suspicion of dengue. Waveform data were processed by the algorithm to calculate each child’s Compensatory Reserve Index, where 1 represents supine normovolemia and 0 represents the circulatory volume at which hemodynamic decompensation occurs. Values between 1 and 0 indicate the proportion of reserve remaining before hemodynamic decompensation.

**Results:**

This case report describes a 7-year-old Thai boy, another 7-year-old Thai boy, and a 9-year-old Thai boy who exhibited signs and symptoms of dengue shock syndrome; all the children had secondary dengue virus infections, documented by serology and reverse transcriptase polymerase chain reaction. The three boys experienced substantial plasma leakage demonstrated by pleural effusion index >25, ascites, and >20 % hemoconcentration. They received fluid administered intravenously; one received a blood transfusion. All three boys showed a significantly low initial Compensatory Reserve Index (≥0.20), indicating a clinical diagnosis of “near shock”. Following 5 days with fluid resuscitation treatment, their Compensatory Reserve Index increased towards “normovolemia” (that is, Compensatory Reserve Index >0.75).

**Conclusions:**

The results from these cases demonstrate a new variation in the diagnostic capability to manage patients with dengue shock syndrome. The findings shed new light on a method that can avoid possible adverse effects of shock by noninvasive measurement of a patient’s compensatory reserve rather than standard vital signs or invasive diagnostic methods.

## Background

Nearly 400 million people are infected with dengue virus (DENV) every year, making dengue the most common mosquito-borne viral disease in humans [1]. The clinical manifestations of DENV infection range from an asymptomatic infection to a debilitating but self-limited illness termed dengue fever (DF), to more severe forms of the disease characterized by increased vascular permeability and plasma leakage, known as dengue hemorrhagic fever (DHF) and dengue shock syndrome (DSS) [2, 3]. Approximately 5 to 10 % of dengue patients experience DHF [3]. The characteristic signs and symptoms of DHF may include abdominal pain, persistent vomiting, petechiae, epistaxis, gingival and gastrointestinal bleeding, microscopic hematuria, thrombocytopenia and, most importantly, hemoconcentration due to increased vascular permeability and third space fluid losses into the pleural and peritoneal cavities. Patients with DHF may progress to develop DSS, evidenced by signs of circulatory failure, including tachycardia, a narrow pulse pressure, hypotension and, in its late stages, lethargy and other mental status changes. Fatality rates among patients with DSS can be 10 % or higher. Fluid resuscitation is essential in DHF/DSS, but overly aggressive fluid administration can exacerbate third space fluid losses, which can lead to pulmonary edema and death when plasma reabsorption occurs. Fatality rates among patients with DSS are typically around 2.5 %, or approximately 22,000 deaths per year [4, 5]. There are no specific antiviral treatments for dengue.

Intravenous fluid therapy in DHF/DSS is guided by repeated physical examinations, frequent vital sign reassessment, serial hematocrits and imaging to assess for intravascular fluid losses and extravascular fluid accumulation (for example, pleural effusion, ascites). The latter strategies are invasive (blood draws) and include exposure to ionizing radiation (chest X-rays), both of which are undesirable in children. Although these strategies help to track volume status, compensatory mechanisms limit their sensitivity until patients are at or near the point of hemodynamic decompensation. Consequently, the capability to measure the integrated response of physiological mechanisms that reflect the sum total of compensation to a relative blood volume deficit could dramatically alter monitoring of patients with dengue and multiple other conditions of hypovolemia. As such, the purpose of the case studies on patients with DHF reported in this paper was to determine if a novel computational algorithm [6–8], originally developed for detecting and monitoring blood loss in combat casualties, could noninvasively indicate the clinical status of children with dengue during the early stages of “shock”, and track their fluid resuscitation status.

## Case presentation

### Patient selection

The cases of a 7-year-old Thai boy, another 7-year-old Thai boy, and a 9-year-old Thai boy were selected to report in this paper. The children presented to the Queen Sirikit National Institute of Child Health (QSNICH), Bangkok, Thailand with clinical suspicion of dengue. None of the three boys met exclusion criteria that included any known chronic condition (for example, liver or renal disease, malignancy, or thalassemia). Informed written consent from a parent or guardian was obtained. This study was approved by the Institutional Review Boards of the QSNICH, Thai Ministry of Public Health, and US Army Surgeon General.

### Study design

Our patients were classified and managed according to the World Health Organization (WHO) 1997 guidelines [2, 9, 10]. Intravenous fluid was initiated by the treating physician at QSNICH when one or more of the following were present: (1) signs suggestive of plasma leakage, that is, rising hematocrit ≥10 % concurrent with thrombocytopenia (platelet count ≤100,000/mm^3^) with poor oral intake; (2) signs of poor peripheral perfusion, including persistent tachycardia, delayed capillary refill (more than 2 seconds), or narrow pulse pressure (≤20 mmHg); and/or (3) need for blood or colloid solution transfusion determined by hematocrit and/or response to fluids administered intravenously.

The following clinical parameters were assessed daily during hospitalization: vital signs (oral temperature, respiratory rate, pulse, and blood pressure collected manually every 3 hours); physical examination findings (presence of ascites, liver enlargement, pleural effusion(s), and hemorrhagic manifestations); tourniquet test result; and complete blood count and serum albumin. Oral intake of fluid and intake of fluid administered intravenously, and urinary output were recorded and totaled every 24 hours. A daily ultrasound examination was performed to assess for ascites, which was read as either present or absent. A chest X-ray was obtained on the day after defervescence (fever day +1) to assess for the presence of pleural effusion, measured as pleural effusion index [9]. When a patient’s temperature was below 38 °C for two consecutive 6-hour periods, then hematocrit determination was conducted every 6 hours by finger stick until stable. In order to track the progression of illness around defervescence and the response to treatment, noninvasive blood pressure waveform data were collected [9]. Patients were hospitalized until they were afebrile, had stable vital signs, and were able to tolerate oral feedings. Additional blood for diagnostic testing was collected on the day of enrollment and approximately 5 to 9 days after discharge.

### Clinical classification

Dengue cases were classified into DF or DHF grade I, II, III, or IV, based on the 1997 WHO case definition [2, 9, 10]. The final case classification of DSS (DHF >III) was retrospectively determined by a physician who was a dengue expert; the dengue expert reviewed the medical records, but did not participate in patient care.

### Laboratory tests

DENV in plasma collected at study entry was detected by a serotype-specific reverse transcriptase polymerase chain reaction (RT-PCR) [11]. Cases were classified as having primary or secondary DENV infection based on the ratio of dengue-specific immunoglobulin G and immunoglobulin M. and by haemagglutination inhibition assay on paired acute and convalescent samples as previously published [9]. The percent hematocrit change was calculated as: (highest hematocrit during hospitalization – hematocrit at convalescence)/(hematocrit at convalescence × 100).

### Noninvasive blood pressure waveform data collection

Continuous noninvasive photoplethysmographic (PPG) waveforms were collected for approximately 15 minutes per day using a Nexfin blood pressure monitor (Edwards Lifesciences, Irvine, CA, USA). The sampling rate was 1000 Hz, exported at a rate of 200 Hz to a computer-based data acquisition software package (WinDAQ; Dataq Instruments, Akron, OH, USA).

### The Compensatory Reserve Index (CRI) algorithm

**Fig. 1:**
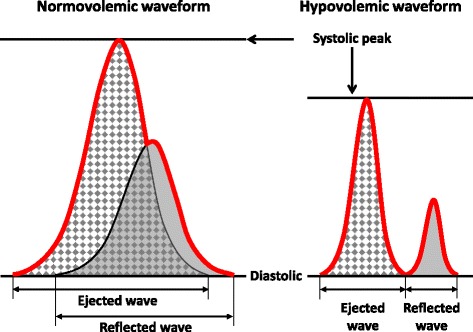
Characteristic features of the arterial ejected and reflected waveforms in states of normovolemia (*left panel*) and central hypovolemia (*right panel*). The *red line* indicates the integrated waveform that would be seen and recorded by an observer. Reproduced from Convertino *et al*. [12]

The compensatory reserve represents a new paradigm for measuring the sum total of all compensatory mechanisms (for example, tachycardia, vasoconstriction, breathing) that together contribute to “protect” against inadequate tissue perfusion during blood loss and other low circulating blood volume states [12]. The physiology is based on the premise that the sum total of all compensatory mechanisms involved in the control of cardiac output (ejected wave) and peripheral vascular resistance (reflected wave) are represented by the entirety of features of the total arterial waveform. Thus, alterations in specific waveform features represent changes in the reserve capacity to compensate for reduced circulating blood volume. This concept is illustrated by the changes in the arterial waveform between “normovolemia” and central “hypovolemia” presented in Fig. 1. Feature extraction and state-of-the-art machine-learning techniques were used to develop a novel computational algorithm that is capable of identifying and monitoring patients during the compensatory phase of central blood volume loss [6–8]. The algorithm was developed at the University of Colorado using data obtained from experiments conducted at the US Army Institute of Surgical Research [6]. In these experiments, lower body negative pressure (LBNP) was used to induce carefully controlled reductions in central blood volume in 100 human participants and validated on 101 different participants [8]. The way the Compensatory Reserve Index (CRI) algorithm for measuring compensatory reserve works is by processing 100 million data points per second in order to evaluate subtle changes in hundreds of specific features of the arterial waveform (outlined in red in Fig. 1). The algorithm used for the current study analyzes how a select group of noninvasive blood pressure waveform features change over time, from normovolemia to decompensation. By monitoring multiple waveform features and knowing how these features change with central blood volume loss, the algorithm determines how near or far an individual may be from the point of decompensation. The algorithm outputs a single value in a beat-to-beat fashion, termed the CRI [7]. Values between 1 and 0 indicate the compensatory reserve of the patient, or the proportion of reserve capacity that remains to compensate for central blood volume loss before the onset of decompensation at a CRI of zero.

### Results

The clinical characteristics of each of our three patients are presented in Table 1. All three children experienced substantial plasma leakage as demonstrated by >25 pleural effusion index, ascites (by physical examination and/or ultrasound) and >20 % hemoconcentration. Clinical evidence of shock included diminished pulse pressure from day 1 to day 2, low systolic blood pressure (Case 2 patient), and/or cold extremities (Table 1). All three children received intravenous fluid during at least the first 2 days of hospitalization and one received a blood transfusion. All three had secondary DENV-2 infections as documented by serology and RT-PCR, and classified as DSS (DHF >III).

**Table 1:** Clinical characteristics of the study patients

Characteristic	Case 1 patient	Case 2 patient	Case 3 patient
Age (years)	7	7	9
Gender	M	M	M
Clinical diagnosis	DHF grade III	DHF grade III	DHF grade III
RT-PCR	DENV-2	DENV-2	DENV-2
Serologic response	Secondary	Secondary	Secondary
Fever day at admission	0	–1	0
Percent hemoconcentration	28	44	39
Pleural effusion index	41	66	26
Clinical evidence of shock			
Systolic BP <80 mmHg	N	Y	N
Pulse pressure <20 mmHg	Y	Y	Y
Cold extremities	Y	Y	Y
Minimum systolic BP (mmHg)			
Study day 1	97	74	97
Study day 2	96	87	93
Minimum pulse pressure (mmHg)			
Study day 1	26	15	29
Study day 2	18	15	15
Fluid intake (ml/kg)			
Study day 1	46	63	25
Study day 2	53	100	86
Study day 3	30	20	46
Study day 4	21	20	35
Transfusion (ml/kg)	4.9 (study day 1)	None	None
Other findings	Ascites, upper GI bleeding	Ascites	Ascites, hemoconcentration, petechiae
Length of hospital stay, days	6	5	5

*BP* blood pressure, *DENV* dengue virus, *DHF* dengue hemorrhagic fever, *GI* gastrointestinal, *M* male, *N* no, *RT-PCR* reverse transcriptase polymerase chain reaction, *Y* yes

**Fig. 2:**
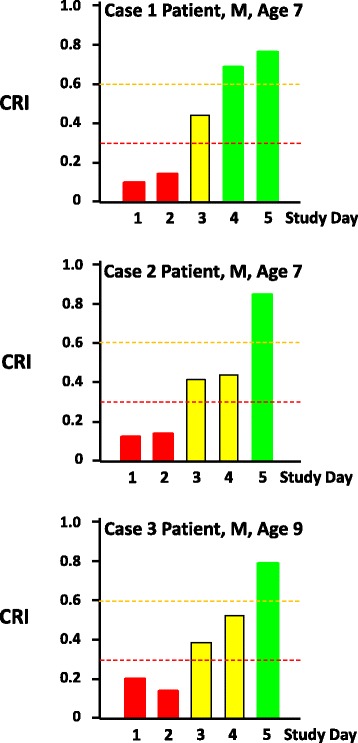
Compensatory Reserve Index values over successive days in three children with dengue shock syndrome. *Red* (unstable), *yellow* (moderately compromised), and *green* (adequate compensation) *bars* indicate the 15 minute average Compensatory Reserve Index for each study day; study day 1 represents the day of study enrollment. *CRI* Compensatory Reserve Index, *M* male

Noninvasive PPG waveform data from the three patients with DSS were de-identified and forwarded to Flashback Technologies, Inc. (Boulder, CO, USA), where the de-identified analog waveform signals were processed by the CRI algorithm. CRI results for these children are shown in Fig. 2. All three children were admitted with significantly low CRI values (≥0.20) on the first and second study days. In all cases, CRI increased on the third study day and the children showed significant improvement towards normovolemia (that is, CRI >0.75) by the end of the period of observation.

## Discussion

In this case series study, a novel algorithm designed to evaluate hemodynamic status based on noninvasive PPG waveform data successfully detected reduced circulating blood volume in three children hospitalized with DSS. The CRI algorithm was originally developed from data collected from experiments conducted on humans ranging in age from 18 to 55 years for continuous, noninvasive, real-time monitoring of blood loss on the battlefield [6–8]. Our findings in the present study using the same algorithm demonstrate that this methodology can be applied to children who present with DSS. The etiology of shock in DSS is mainly related to plasma leakage leading to reduced intravascular volume [2]. The similarities of this process to hemorrhagic shock may therefore have contributed to the success of the CRI algorithm in this patient population. Based on the successful translation of measuring the compensatory reserve in young children (7 to 9 years of age), and the underlying physiological concept of CRI, we hypothesize that the algorithm may translate accurately to older patients despite possible age-related differences in compensatory responses. Separate clinical studies are currently being conducted to test whether the algorithm will apply equally well in other disease processes, such as sepsis, as well as older patient populations.

All three patients had an abnormally low CRI at initial presentation, consistent with the clinical evidence for diagnosis of shock (Table 1). CRI values remained low for several days despite fluid administration and, in one patient, blood transfusion. CRI values gradually improved and normalized by day 4 in one patient and by day 5 in two patients (Fig. 2). In this regard, it is critical to avoid hypervolemia in critically ill patients by targeting “ideal” filling volume with the goal of minimizing or eliminating the possibility of creating “third spacing” and interstitial edema [13–15]. The progressive restoration of CRI during resuscitative interventions in the present case studies supports the concept that the “individualized” assessment of compensatory reserve may provide a patient-centric approach to goal-directed resuscitation and avoid clinical complications of potential over-resuscitation.

The period of plasma leakage in DHF occurs within a 24 to 48 hour “critical phase” around the time of defervescence [16]. The phenomenon of plasma leakage is evident in the fluid volumes received by the three patients during the first 3 days and further supported by serial ultrasound evaluations. Intravenous fluid resuscitation in DSS is guided by clinical assessment of peripheral perfusion and serial hematocrit determinations. Overly aggressive fluid administration is avoided to minimize the late complications of intravascular volume overload when the extravascular fluid is resorbed [2]. The persistently low CRI values that we observed in the first few days following defervescence were likely due to some combination of plasma leakage and the conservative approach to fluid therapy. Data reported from controlled hemorrhage and blood replacement experiments conducted on human participants support the findings of the present case studies that the CRI can provide a more sensitive and specific indicator of intravascular volume than the clinical assessments (that is, standard vital signs) used to guide fluid therapy in DSS [17–19]. A clinical trial comparing standard therapy to CRI-directed fluid resuscitation will be required to substantiate this hypothesis.

The three patients in our case series were managed according to published guidelines for dengue case management [2, 9, 10]; this represents the standard approach used at our hospital. Intravenously administered fluid therapy and blood transfusion (the latter given in case 1 only) are detailed in Table 1. The CRI algorithm was applied retrospectively to the analysis of PPG waveforms that were collected in the course of hemodynamic monitoring. The results of this analysis, which are not cleared by the US Food and Drug Administration (FDA) for clinical use, were not provided to the clinicians nor used to guide clinical care. As such, our report places the CRI data in the context of routine clinical care to illustrate the potential impact of the technology, with the caution that the actual impact of using the CRI to guide patient management would need to be validated in the context of a clinical study done under rigorous standards. Case 1 was discharged on day 6, and cases 2 and 3 were discharged on day 5. All three patients were seen in clinic 4 days after discharge; all recovered fully with no sequelae.

The recognition that indicators of blood loss can be linked to characteristic features of the photoplethysmogram waveform is not new. However, there are distinct advantages of the technological approach of PPG waveform analysis used in the CRI algorithm compared to other technologies that employ arterial pulse contour analysis for noninvasive estimates of cardiac output (for example, PiCCO®, USCOM®). The measurement of cardiac output alone fails to provide additional critical information about other compensatory mechanisms (for example, peripheral vascular control) for complete and accurate assessment of patient status. Although proven to be responsive to reductions in central blood volume (that is, sensitivity), the primary limitation of these techniques is that they are based on population averages from clinical trials and fail to account for variation in an individual patient’s ability to compensate and subsequently tolerate (that is, low specificity) severe reductions in circulating blood volume [12]. As such, the relatively poor specificity produced by these population-based algorithms fails to provide early prediction of hemodynamic decompensation to progressive reduction in central blood volume in those patients at greatest risk for developing hemorrhagic shock. In contrast, measurement of the compensatory reserve using the CRI algorithm has now been validated in several studies of experimentally controlled central hypovolemia [8, 19] and clinical conditions of severe hemorrhage [20].

Through high-speed computing processes that combine machine learning with feature extraction, we have developed an algorithm capable of integrating all of the physiological compensatory mechanisms and the changes in their status and features in response to compromised circulating blood volume, which gives the CRI its unique individual specific predictive capability to assess one’s capacity to compensate. Unlike previous monitoring algorithms that are based on average responses of clinical populations, the CRI represents the first diagnostic technology for the assessment of compensatory status in an individual patient. Each patient’s compensatory reserve is correctly estimated because the machine-learning capability of the algorithm accounts for compromised circulating blood volume. It “learns” and “normalizes” the totality of compensatory mechanisms based on the individual’s arterial waveform features, and thus provides “precision medicine” that is patient centric for accurate diagnosis and resuscitative treatment of the “individual” patient.

## Conclusions

The present study provides preliminary evidence of the value of the CRI algorithm in the management of clinical conditions of central hypovolemia such as those associated with dengue. Although the results should be interpreted with caution given the small sample size and lack of comparative data from patients with milder forms of dengue or other febrile illnesses, they were strikingly consistent in all three patients. Balancing the limitations of the current study are the significant potential advantages of this technology. The CRI algorithm provides a continuous, beat-to-beat objective interpretation of PPG waveform data; the interpretation does not require specialized expertise [6, 7]. The CRI algorithm does not require baseline or convalescent data; it can be acquired in real-time during any clinical encounter [7, 8]. The machine-learning capability of the CRI algorithm allows for identification of a specific patient, and thus provides individualized medicine. Lastly, the CRI algorithm can interpret waveform data from noninvasive monitoring devices that are relatively widely available, such as portable pulse oximeters, which could be produced at low cost [7]. Thus, rapid translation of the present findings to low-resource settings is highly feasible, and would provide information that is not obvious to clinicians and other health care providers. Since delayed or inappropriate medical care is a major factor in dengue-related morbidity and mortality and other clinical conditions of reduced central blood volume (for example, hemorrhage), our data support the notion that the CRI algorithm is a promising technology with the potential for making a significant public health impact.

## Abbreviations

CRI, compensatory reserve index; DENV, dengue virus; DF, dengue fever; DHF, dengue hemorrhagic fever; DSS, dengue shock syndrome; LBNP, lower body negative pressure; PPG, photoplethysmographic; QSNICH, queen sirikit national institute of child health; RT-PCR, reverse transcriptase polymerase chain reaction; USAISR, US army institute of surgical research; WHO, world health organization
